# The relationship between nurses’ psychological resilience and job satisfaction during the COVID-19 pandemic: a descriptive-analytical cross-sectional study in Iran

**DOI:** 10.1186/s12912-023-01310-z

**Published:** 2023-04-25

**Authors:** Parvin Mangolian Shahrbabaki, Parniya Abolghaseminejad, Leyla Ahmadi lari, Somayeh Zeidabadinejad, Mahlagha Dehghan

**Affiliations:** 1 Nursing Research Center, Kerman University of Medical Sciences, Kerman, Iran; 2 Department of Public Health, Sirjan School of Medical Sciences, Sirjan, Iran; 3 Department of Nursing, School of Nursing, Larestan University of Medical Sciences, Larestan, Iran; 4 Sirjan School of Medical Sciences, Sirjan, Iran

**Keywords:** Resilience, Psychological, Job satisfaction, Nurses, Iran

## Abstract

**Background:**

Job satisfaction and factors affecting them are among the most important social issues. Resilience has a moderating role in the relationship between stress and diseases, so it can affect a person's job satisfaction because it enables a person to deal with adverse conditions. This study aimed to investigate the relationship between nurses’ psychological resilience and job satisfaction during the COVID-19 outbreak.

**Methods:**

This descriptive-analytical cross-sectional study (2022) used convenience sampling to select 300 nurses. The Connor and Davidson Resilience Scale and Minnesota Satisfaction Questionnaire were used to collect data. The data were then analyzed with SPSS 22 and statistical methods (Independent t-test, Analysis of Variance, Pearson correlation coefficient, and Multiple linear regression).

**Results:**

The study results showed a positive and poor relationship between resilience, some of its dimensions (trust in individual instincts, tolerance of negative affect (*p* = 0.006), positive acceptance of change and secure relationships (*p* = 0.01), spiritual influences (*p* = 0.04)) and job satisfaction (*p* < 0.001). In other words, nurses’ high level of resilience increased their job satisfaction and vice versa.

**Conclusions:**

Enhancing the resilience of frontline nurses during the COVID-19 pandemic improved their job satisfaction and affected care provided by them. Nurse managers can control nurses’ resilience and offer interventions that would strengthen it, especially at crises.

## Introduction

The healthcare system has encountered an unusual pressure and significant challenges during the COVID-19 outbreak [1]. Frontline nurses may experience stress and anxiety because they often witness patients’ suffering [2], which has a significant impact on their mental and emotional well-being [3]. Literature has suggested following sources of anxiety among nurses: prolonged working hours, lack of personal protective equipment (PPE), concern about transmitting the virus, and the stress of making ethical decisions about prioritization of care [4, 5]. Such stressors may have prolonged effects on their job satisfaction and work performance, leading to their turnover intention [1].

Job satisfaction is critical for nurses caring for patients during a pandemic [6] and many factors affect it such as individual characteristics, working environment, salary, recognition, and career advancement, which in turn influence their decisions to stay or quit their jobs [7, 8]. In such difficult circumstances, resilience is a vital requirement for nurses’ endurance [2].

Resilience is the ability to cope successfully with stressful events [9] and reduces the effects of workplace stressors, so it is an effective way to enhance employees’ well-being [10–12]. High resilience has a close relationship with nurses’ reduced burnout and turnover [10–13]. Research on resilience has focused on its role in improving the quality of care and enhancing patient satisfaction [9]. Factors affecting resilience include physiological factors (e.g., sympathetic nervous system), internal factors (e.g., self-efficacy, inner wisdom), external factors (e.g., clinical settings, social network), and demographic variables (e.g., age, years of experience) [13, 14]. Several studies highlighted the significant correlations between nurses’ well-being, resilience, and turnover [10–13]. Recent evidence revealed a positive association between resilience, job satisfaction, job retention, general well-being, and social support [15]. Occupational stressors and high workload have a negative effect on nurses’ physical, mental, and professional well-being and lead to poor practice, low job satisfaction, and high turnover [16], an additional workload for remaining nurses, and a vicious circle for more burnout. Violence exacerbates this circle, while resilience reduces it.

To help healthcare personnel and to improve the quality of services to patients, the present study examined the impact of resilience on job satisfaction in nurses during the COVID-19 outbreak. Since nurses undergo psychological pressures and many stressors, they must improve their resilience to overcome challenges, unpleasant events and conditions and create favorable working conditions. We hope that our results control and prevent job abandonment during the COVID-19 outbreak, manage job satisfaction and improve health level and practice in the personnel. The present study aimed to determine the relationship between nurses’ psychological resilience and job satisfaction during the COVID-19 outbreak in 2022.

## Method

### Study type

The current study was descriptive-analytical and cross-sectional. The statistical population of this research was nurses working in Imam Reza hospital in Sirjan in 2022. All eligible nurses were selected by convenience sampling method.

### Sample and sampling

Inclusion criteria: nurses who were working in different departments during the COVID-19 outbreak, signed the informed consent form and were willing to participate in the study.

Exclusion criterion: nurses who failed to answer more than a third of the questions. The sample size was calculated according to a similar study conducted by Amini [17]. According to the standard deviation equal to 16.26 of the psychological resilience score in Amini's study, 5% error and 2% accuracy, 251 nurses were calculated, but 300 participants were included in the study for better estimation and the possibility of incomplete answers.

### Research tools

Demographic information questionnaires, the Connor and Davidson Resilience Scale, and the Minnesota Satisfaction Questionnaire were used to collect information.

Demographic information questionnaire included age, sex, marital status, level of education, work experience, employment status, type of ward and answers to these questions: have you ever been infected with coronavirus? Have you lost someone close to you due to coronavirus infection?

#### Connor and Davidson Resilience Scale

The Connor and Davidson Resilience Scale contains 25 items on a five-point Liker scale ranging from zero (not true at all) to four (true nearly all of the time). These ratings result in a number between 0–100, and higher scores indicate higher resilience; the mean score of resilience is 50 [18]. Validity (factor analysis method and convergent and divergent validity) has been verified by the creators of the test in normal and at-risk groups, and the reliability coefficient obtained from the retest method in a 4-week interval was 0.87 [19]. This instrument had good validity and reliability and was standardized and validated by Mohammadi (2005). Cronbach's alpha method was used to determine the reliability and the reliability coefficient was 0.89. To determine its validity, we calculated the correlation between each score and the total score, except for item 3, and obtained coefficients of 0.41–0.64. The scale items were then analyzed by the principal components analysis. Before extracting the components according to the correlation matrix, we calculated them using the KMO index and Bartlett test of sphericity. The KMO value was 0.87 and the chi-square value in Bartlett’s test of sphericity was 5556.28, both of which explained the adequacy of evidence for factor analysis calculation [20].

#### Minnesota Satisfaction Questionnaire

The Minnesota Satisfaction Questionnaire contains 19 items on a five-point Likert scale (strongly disagree, disagree, undecided, agree, and strongly agree). The scores of 19–38 indicate poor job satisfaction, scores of 38–57 indicate moderate job satisfaction, and scores more than 57 indicate very good job satisfaction. In general, the reliability coefficients obtained were high. For the intrinsic satisfaction scale, the coefficients ranged from 0.84 (for the two assembler groups) to 0.91 for engineers. For the extrinsic satisfaction scale, the coefficients varied from 0.77 to 0.82 (for engineers and machinists). For the general satisfaction scale, the coefficients varied from 0.87 (for assemblers) to 0.92 (for engineers). Median reliability coefficients were 0.86 for intrinsic satisfaction, 0.80 for extrinsic satisfaction, and 0.90 for general satisfaction [21]. Various studies reported favorable validity and reliability for this questionnaire, which used to measure satisfaction with nursing, management, production, service and education. The guide of the main version of the questionnaire reported the reliability coefficient of 0.89 using the retest method and confirmed the content validity [22]. Hajibabaee reported reliability of 0.82 and confirmed its validity using the content validity method [23]. Hadizadeh Talasaz confirmed reliability of 0.86 using the Cronbach's alpha coefficient and its validity using face and content validity [24].

### Data collection

After receiving the code of ethics and an official letter of introduction from Kerman University of Medical Sciences, the researcher went to Imam Reza hospital in Sirjan, explained the study objectives, coordinated with the relevant authorities, and then started sampling. The researcher used convenience sampling method to select eligible nurses, explained them the study objectives, and received their written informed consent. The nurses were asked to answer the questions of the questionnaires during their break or at the end of their shifts and then put the questionnaires in the place specified in the ward (rest room). The researcher usually visited the relevant department once every two days to collect the questionnaires and continued this action to reach the desired sample size (300). The nurses could contact the researcher if they had any questions or doubts about the questions in the questionnaire. All participants were assured that the questionnaires would be anonymous and their information would be confidential.

### Data analysis

Frequency, percentage, mean and standard deviation were used to describe the study variables. Skewness, Kurtosis, and Kolmogrov-Smirnov test were used to check the normal distribution of the data. Levene’s test was used to check the equality of variances. As parametric condition was fulfilled, analysis of variance, independent t-test and Pearson correlation coefficient were used for bivariate analyses. Multiple linear regression was used to investigate the predictors of resilience. SPSS26 was used for data analysis. The significance level was considered < 0.05.

## Results

The mean age of the samples was 32.06 ± 5.56 years (minimum = 24 and maximum 46). The mean work experience was 6.39 ± 4.18 years. The majority of the samples were female, married, with a bachelor's degree, and a work experience of less than 5 years. Most of the samples reported a history of COVID-19 twice. Most of them were vaccinated three times against COVID-19 (Table 1).

**Table 1:** The relationship between demographic characteristics, resilience and job satisfaction

Variable	Frequency (%)	Resilience		Statistical test (*P*-value)	Job satisfaction		Statistical test (*P*-value)
		Mean	SD		Mean	SD	
Age (yr)				F = 1.29 (0.28)			F = 0.48 (0.62)
24–30	146 (48.7)	58.20	4.41		39.38	3.78	
30.1–35	65 (21.7)	57.38	4.56		39.02	3.99	
> 35	89 (29.7)	58.60	5.09		39.63	3.87	
Sex				t =—0.23 (0.82)			F = 0.48 (0.62)
Male	95 (31.7)	58.23	4.48		39.75	3.71	
Female	205 (68.3)	58.10	4.56		39.20	4.12	
Marital status				t =—0.30 (0.76)			t = 0.96 (0.34)
Single	165 (55.0)	58.07	4.50		39.57	3.87	
Married	135 (45.0)	58.23	4.86		39.14	3.80	
Education level				t = -0.31(0.75)			t = -1.47(0.14)
Bachelor’s degree	252(84.0)	58.10	4.68		39.23	3.71	
Master’s degree	48(16.0)	58.33	4.61		40.13	4.46	
Work experience				F = 1.37(0.26)			F = 0.25(0.78)
1–5	154(51.4)	58.12	4.49		39.45	3.84	F
5.1–10	88(29.3)	57.65	4.58		39.14	3.79	
> 10	58(19.3)	58.95	5.16		39.53	4.0	
Employment				F = 0.31(0.87)			F = 0.40(0.81)
Hired	78(26.0)	58.63	4.85		39.67	3.81	
Contract recruiter-1^a^	94(31.4)	57.91	4.73		39.48	3.94	
Contract recruiter-2^b^	49(16.3)	58.14	4.15		38.86	3.66	
Committed^c^	54(18.0)	57.91	4.77		39.41	3.82	
Contract recruiter-3^d^	25(8.3)	57.96	4.73		39.04	4.22	
Workplace				F = 0.37(0.90)			F = 0.68(0.67)
Emergency	26(8.7)	57.42	4.98		39.04	5.30	
CCU	34(11.3)	58.21	4.26		39.88	3.94	
COVID-19 ICU	55(18.3)	58.60	4.74		38.78	3.48	
ICU General	40(13.3)	58.15	4.90		40.03	3.81	
COVID-19 Outpatient Treatment	54(18.0)	57.76	4.90		39.59	3.84	
COVID-19 hospitalization	61(20.3)	57.98	4.59		39.44	3.43	
Coronavirus-related care	30(10.0)	58.83	4.32		38.80	3.89	
History of COVID-19 infection				t = -1.13(0.26)			t = 0.79(0.43)
Yes	261(87.0)	58.02	4.66		39.44	3.86	
No	39(13.0)	58.92	4.66		38.92	3.79	
Frequency of getting COVID-19 infection				F = 1.96 (0.12)			F = 0.21(0.89)
0	39(13.0)	58.92	4.66		38.92	3.79	
1	57(19.0)	57.93	4.68		39.40	4.27	
2	166(55.3)	57.72	4.51		39.47	3.41	
3	38(12.7)	59.50	5.08		39.39	5.01	
Relatives’ death				t = 1.88(0.06)			t = 1.07(0.29)
Yes	113(37.7)	58.79	4.66		39.68	4.01	
No	187(62.3)	57.75	4.63		39.19	3.74	
Number of vaccinations				t = -0.55(0.58)			t = -0.49(0.62
2	136(45.3)	57.98	4.83		39.26	3.78	
3	164(54.7)	58.27	4.52		39.48	3.91	

^a^Annually contracted with payment less than hired nurses

^b^Annually contracted with payment similar to hired nurses

^c^It is obligatory to work for government for two years at a lower rate of pay

^d^Contract recruiter are people that are hired by intermediary companies (manpower supply companies) for government organizations (government agencies) or private companies

F = analysis of variance t = Independent t-test

The mean resilience score was 58.14 ± 4.66, which was higher than the midpoint of the questionnaire [15]. The mean per item of all dimensions was close to each other (Table 2). The resilience of all samples was moderate. The mean job satisfaction score was 39.38 ± 3.85 (Table 2). Job satisfaction of 40.3% (*n* = 121) of the nurses was poor and that of 59.7% (*n* = 179) was moderate.

**Table 2:** Description of resilience, job satisfaction and their dimensions in nurses

Variable		Minimum	Maximum	Mean	SD	Mean per item
Resilience	Personal competence	14	24	18.83	2.03	2.35
	Trust in one’s instincts, tolerance of negative affect	10	21	15.91	2.08	2.27
	Positive acceptance of change and secure relationships	6	15	11.72	1.70	2.34
	Control	4	9	7.0	1.25	2.33
	Spiritual influences	2	6	4.69	0.86	2.34
	Total	45	71	58.14	4.66	2.04
Job satisfaction	Pay	4	9	6.11	0.85	2.13
	Career type	5	12	8.52	1.51	2.11
	Development opportunities	3	9	6.34	1.31	2.10
	Organizational atmosphere	2	6	4.21	1.06	2.17
	Leadership style	5	12	8.67	1.44	1.84
	Working condition	3	9	5.52	1.49	2.04
	Total	28	49	39.38	3.85	

We found a positive and poor relationship between nurses’ resilience, some of its dimensions (trust in individual instincts, tolerance of negative affect, positive acceptance of change and secure relationships, and spiritual influences) and job satisfaction, with higher resilience indicating higher job satisfaction and vice versa (Table 3). We observed no significant relationship between resilience, age, sex and marital status, as well as between job satisfaction, age, sex and marital status (Table 1).

**Table 3:** The relationship between resilience, its dimensions, and job satisfaction in nurses

Variable	Job satisfaction	
	Pearson coefficient	*p*-value
Resilience		
Personal competence	0.10	0.08
Trust in one’s instincts, tolerance of negative affect	0.16	0.006
Positive acceptance of change and secure relationships	0.15	0.01
Control	0.06	0.27
Spiritual influences	0.12	0.04
Total	0.21	< 0.001

We further tested multiple regression models with stepwise method to explore how demographic variables could predict resilience. All variables (frequency of getting COVID-19, relatives’ death and job satisfaction) with a *P* value of < 0.2 in bivariate analysis (Tables 1 and 3) were included in the model. Only job satisfaction remained in the model and predicted 4% of the variance of resilience (*p* < 0.05) (Table 4).

**Table 4:** The multiple regression analysis summary for resilience among the participants

**Variable**	**B**	**SE**^a^	**β**	**T**	**P**	**95% CI** **Lower**	**95% CI** **Upper**	**Adjusted R**^**2**^
Constant	48.19	2.72		17.75	< 0.001	42.85	53.54	0.04
Job satisfaction	0.25	0.07	0.21	3.68	< 0.001	0.12	0.39	

^a^Standard error; Data were presented as multiple regression analysis. Only significant results were shown; *CI* Confidence intervals for B

## Discussion

The present study aimed to investigate the relationship between nurses’ psychological resilience and job satisfaction during the COVID-19 outbreak in 2022. Our results indicated moderate resilience level among nurses. Some other studies supported our results and indicated moderate levels of resilience among nurses during the COVID-19 pandemic [25–27]. Roberts et al. (2021) in the UK found that the nurses’ resilience score was moderate during the COVID-19 pandemic [28]. Luceño-Moreno et al. (2020) in Spain reported moderate levels of resilience among healthcare providers during the COVID-19 pandemic [29]. Yusefi et al. (2021) in Iran demonstrated moderate levels of resilience among nurses during the COVID-19 outbreak [30]. Profession, personal characteristics and environmental and social factors all affect resilience [28, 31]. Resilience is effective in maintaining mental health and preventing stressful events such as the COVID-19 pandemic [32].

Most of the nurses in our study had moderate job satisfaction. Bayer et al. (2021) agreed with us and found that Turkish nurses had moderate job satisfaction during the COVID-19 pandemic [33]. Savitsky et al. (2021) also indicated moderate/high job satisfaction during the COVID-19 outbreak [34]. Labrague et al. (2020) and Heidari et al. (2022) disagreed with us and suggested poor job satisfaction among nurses during the COVID-19 outbreak [35, 36]. Yu et al. (2020) also concluded that job satisfaction was high among the frontline nurses during the COVID-19 pandemic [37]. It is noteworthy that different nursing management systems and working conditions in different medical centers can affect nurses' job satisfaction.

Nurses are at risk of infection with the COVID-19 due to their direct contact with patients. Nurses' job satisfaction reduced when the number of patients admitted to the COVID-19 wards increased during the pandemic [38]. Nurse managers should increase the number of nurses and reduce their workload to enhance job satisfaction among nurses working in the COVID-19 wards [36].

We found a positive relationship between nurses' resilience, some of its dimensions and job satisfaction, with higher resilience indicating higher job satisfaction of nurses. Alameddine et al. in Lebanon (2021) supported our results and reported a direct relationship between nurses’ resilience and job satisfaction during the COVID-19 pandemic [2]. The studies conducted before the COVID-19 outbreak also showed a relationship between resilience and job satisfaction in nurses. Piotrowski et al. (2022), Bernard (2021), Brown et al. (2018), Zheng et al. (2017) and Hudgins (2015) confirmed the positive effect of resilience on job satisfaction [39–43]. Studies in the United States [41, 43, 44], Singapore [42], Turkey [45] and Pakistan [46] also showed a positive relationship between nurses’ job satisfaction and resilience because resilience helped nurses to keep their practice effective, overcome their problems, and improve their mental health and well-being. Resilience helps people perceive stressful situations as challenges and consider failures as normal outcomes [39]. Hou et al. (2020) reported a significant effect of job satisfaction and resilience on job performance [47]. Zhao et al. (2021) found an indirect effect of resilience on job abandonment through job satisfaction and social support [48].

Resilience causes individuals to restore their well-being after traumatic experiences. Although, stress has a negative effect on resilience, it is a universal resource that ensures job performance regardless of the pandemic [15]. Resilient nurses cope with problems at work and strengthen themselves to bounce back from a negative outlook [49].

Ghandi et al. (2017) indicated a direct effect of resilience on job satisfaction among Iranian nurses [50]. Highly resilient individuals have better health, self-esteem and are less stressed, which reduce their risk of depression. Resilience causes control over work, creates job satisfaction, and increases efficiency and productivity [50].

As resilience has a significant relationship with job satisfaction, we can use interventions to enhance nurses’ resilience, job satisfaction, and ultimately their working conditions [51]. Resilience workshops could also help nurses adapt to challenging circumstances [52].

## Limitations

This study had several limitations. Although, the researchers tried to convince nurses that their participation had no influence on their occupational position, they encountered a social desirability bias. Although, nurses’ experience at the target hospital exemplifies that of nurses across the healthcare systems, we can generalize the results only to hospitals with a similar context. The data were collected when the frontline nurses were receiving public material and moral support, which might have affected their responses.

## Conclusion

We found a positive correlation between nurses’ resilience and job satisfaction. High level of resilience has a significant positive effect on providing care for patients and would improve their retention in emergencies. Nurses must enhance their resilience and well-being to balance work and life responsibilities. Therefore, we suggest more focus on interventions aimed at improving nurses' resilience in work environments in future researches.

## Data Availability

All data generated or analysed during this study are included in this published article.
